# Polyfluorene-Based Multicolor Fluorescent Nanoparticles Activated by Temperature for Bioimaging and Drug Delivery

**DOI:** 10.3390/nano9101485

**Published:** 2019-10-18

**Authors:** Marta Rubio-Camacho, Yolanda Alacid, Ricardo Mallavia, María José Martínez-Tomé, C. Reyes Mateo

**Affiliations:** Instituto de Investigación Desarrollo e Innovación en Biotecnología Sanitaria de Elche (IDiBE), Universidad Miguel Hernández de Elche (UMH), 03202 Elche, Alicante, Spain; marta.rubioc@umh.es (M.R.-C.); yoli2395@gmail.com (Y.A.); r.mallavia@umh.es (R.M.)

**Keywords:** multifunctional fluorescent nanoparticles, conjugated polyelectrolytes (CPEs), thermosensitive liposomes (TSLs), bioimaging, drug carrier, release experiments

## Abstract

Multifunctional nanoparticles have been attracting growing attention in recent years because of their capability to integrate materials with different features in one entity, which leads them to be considered as the next generation of nanomedicine. In this work, we have taken advantage of the interesting properties of conjugated polyelectrolytes to develop multicolor fluorescent nanoparticles with integrating imaging and therapeutic functionalities. With this end, thermosensitive liposomes were coated with three recently synthesized polyfluorenes: copoly-((9,9-bis(6′-*N,N,N*-trimethylammonium)hexyl)-2,7-(fluorene)-alt-1,4-(phenylene)) bromide (HTMA-PFP), copoly-((9,9-bis(6′-*N,N,N*-trimethylammonium)hexyl)-2,7-(fluorene)-alt-4,7-(2- (phenyl)benzo(d) (1,2,3) triazole)) bromide (HTMA-PFBT) and copoly-((9,9-bis(6′-*N,N,N*- trimethylammonium)hexyl)-2,7-(fluorene)-alt-1,4-(naphtho(2,3c)-1,2,5-thiadiazole)) bromide (HTMA-PFNT), in order to obtain blue, green and red fluorescent drug carriers, respectively. The stability, size and morphology of the nanoparticles, as well as their thermotropic behavior and photophysical properties, have been characterized by Dynamic Light Scattering (DLS), Zeta Potential, transmission electron microscope (TEM) analysis and fluorescence spectroscopy. In addition, the suitability of the nanostructures to carry and release their contents when triggered by hyperthermia has been explored by using carboxyfluorescein as a hydrophilic drug model. Finally, preliminary experiments with mammalian cells demonstrate the capability of the nanoparticles to mark and visualize cells with different colors, evidencing their potential use for imaging and therapeutic applications.

## 1. Introduction

Nanomedicine is an emergent area which results from the application of nanotechnology to medicine. Research in this field has experienced rapid growth during the last decade, extending its applications in bioimaging, disease treatment and diagnosis. A number of nanoformulations for diagnostics and therapeutics have been approved for use in humans, and even more are currently under investigation [1,2,3]. An important trend in this field is the development of multifunctional nanoplatforms integrating different properties, such as imaging and therapeutic functionalities, in one entity [4,5]. In this regard, a large variety of biocompatible materials including lipids, proteins, carbon and quantum dots, synthetic polymers, fluorophores, dendrimers or metallic nanoparticles have been coupled and organized in nanostructures forming vesicles, micelles, nanorods, dendrimers and more, to be used as nanomedical vehicles in theragnostic applications [6,7,8].

The main component of these nanostructures is the carrier, which is responsible for transporting the drug and releasing it. Since their discovery in the mid-1960’s, liposomes, which are lipid vesicles formed by one or more concentric lipid bilayers surrounding an aqueous core, have been considered to be the most successful nanocarriers for drug delivery, especially in anticancer chemotherapy [9,10]. This success is mainly due to the numerous advantages they offer. On one hand, liposomes are highly versatile, allowing the incorporation of hydrophobic drugs in the lipid bilayer or hydrophilic drugs in the aqueous core, as well as surface modifications in order to control their interactions with biological targets. On the other hand, the lipid membrane can behave as a barrier contributing to protect the encapsulated drug from degradation, thus prolonging its half-life in the bloodstream. In addition, due to the enhanced permeability and retention (EPR) phenomenon displayed by tumor tissues in comparison with normal tissues, liposomes can selectively accumulate in the tumor, enhancing efficacy and minimizing adverse side effects [11,12,13]. 

The release of drugs from the liposomal formulation is usually very low; nevertheless, the fact that the physical properties of the lipid membrane respond to a wide range of internal and external stimuli (temperature, pH, light, pressure, ions, magnetic field, etc.) can be used to selectively release the drug in a controlled way [14]. One well-established approach for triggering the encapsulated drug release, benefits from the differences in the permeability of the lipid membrane between the gel and fluid phase. In the gel phase, lipids are closely packed, with the acyl chains extended, and there is little lateral diffusion and low permeability. On the contrary, in the fluid phase, the acyl chains are more kinked, packing is lost, and the permeability is relatively high. The gel–fluid phase transition occurs cooperatively at the transition temperature (T_m_), so it is possible to increase the permeability of the lipid bilayer by increasing the temperature beyond T_m_, allowing the release of entrapped drugs [15,16]. The permeability is additionally increased just at this temperature, as a consequence of the coexistence of membrane areas in both phases [17].

Liposomes which release their contents at specific temperatures are called thermosensitive liposomes (TSLs) [18,19]. Among the different TSLs reported in the literature, the most clinically preferable are those having their T_m_ between 39–43 °C (mild hyperthermia), because these temperatures improve drug uptake, increase tumor perfusion and rend cancer cells temporarily sensitive to other treatments [20,21]. Typical TSLs have been mostly prepared from 1,2-dipalmitoyl-sn-glycero-3-phospho-rac-(1-glycerol) sodium salt (DPPG) and 1,2-dipalmitoyl-sn-glycero-3-phosphocholine (DPPC) derivatives, alone or combined with other lipids or polymers, due to its drug encapsulation capacity, excellent biodegradability and because their T_m_ occurs at 41–42 °C [22,23]. In the hyperthermia treatment, the tumor is locally heated to 40–43 °C for a defined period of time. The drug circulating in the bloodstream is safely entrapped in the TSLs because the liposomal membrane is in the gel phase, but once it reaches the heated tumor, the phase transition takes place and the drug is released, generally by passive transfer across the membrane according to a concentration gradient [24]. 

Incorporation of reporter groups—such as fluorescent components—in drug delivery carrier systems is of great interest in the fabrication of multifunctional nanoplatforms since they can act as probes for bioimaging and labelling (diagnosis) while monitoring the pathway concerning the drug, providing information from its final location [25,26]. During the last decades, these fluorescent components have been usually small organic fluorophores, fluorescent proteins and inorganic quantum dots (QDs) [27]. In contrast to organic dyes and fluorescent proteins, QDs do not suffer from photobleaching, self-quenching or chemical degradation and show unique properties such as broad absorption bands, high molar extinction coefficients, narrow emission peaks and large Stoke shifts. In addition, QDs made up of the same material have distinct emission wavelengths, depending on their size, shifting to the red as the size of QDs increases. These properties allow to simultaneously excite mixed QDs populations with different emission wavelengths at a single wavelength, facilitating multicolor fluorescence imaging [28]. Nonetheless, the potential cytotoxicity risk associated with the chemical composition of QDs (cadmium, selenium, tellurium, etc.) remains the major limitation for using these fluorescent nanoparticles in biological approaches [29,30,31]. To overcome this concern, it is necessary to develop safe and more efficient fluorescent carriers which, in addition to encapsulating the drug, incorporate new fluorescent materials with improved characteristics, such as nontoxicity, stability, high sensitivity, etc.

In comparison with common organic dyes, fluorescent proteins and QDs, conjugated polyelectrolytes (CPEs) have unique physiochemical properties. These materials are polymers with highly electron-delocalized backbones, containing ionic side groups which facilitate their water solubilization. CPEs have the optoelectronic properties from their neutral counterpart conjugated polymers (CPs), showing high-fluorescent quantum yields, broad absorption and emission spectra, large Stokes shift, good photostability, and more efficient intramolecular/intermolecular energy transfer than common organic dyes and fluorescent proteins, while having the advantage of being more biocompatible than QDs [32]. Contrary to CPs, CPEs display the typical physicochemical behavior of polyelectrolytes in aqueous solvents, allowing the coupling with different biological systems via electrostatic interactions [33]. In addition, CPEs have easily tunable side chains for bio-conjugation with several recognition elements and show high versatility in their synthesis, enabling fine-tuning of their absorption and emission bands through backbone modification [34]. Given these properties, CPEs have been successfully applied for detection of a wide range of biological and chemical molecules, but also as novel fluorescent probes for bioimaging, and exhibit enormous potential for therapeutic applications and/or diagnostics [35,36,37,38]. 

**Scheme 1:**
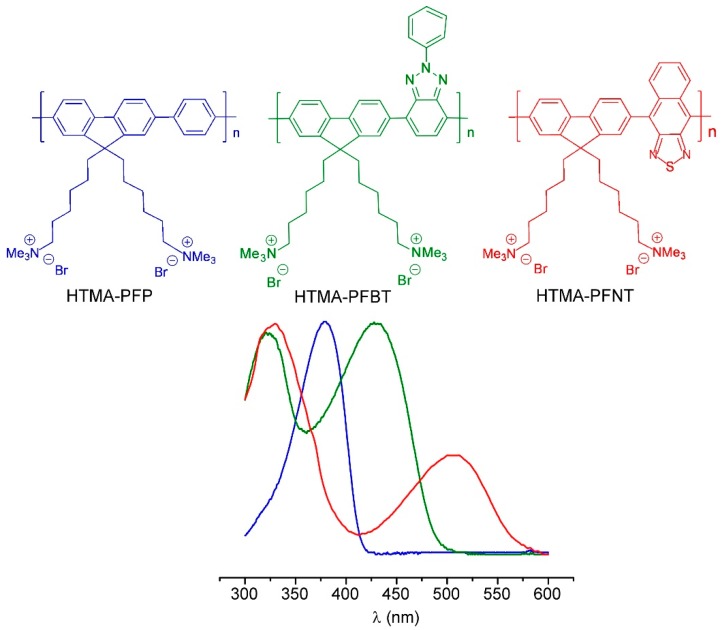
Chemical structures (**upper part**) and normalized absorption spectra (**bottom part**) of conjugated polyelectrolytes (CPEs) copoly-((9,9-bis(6′-*N,N,N*-trimethylammonium)hexyl)-2,7- (fluorene)-alt-1,4-(phenylene)) bromide (HTMA-PFP) (in blue), copoly-((9,9- bis(6′-*N,N,N*-trimethylammonium)hexyl)-2,7-(fluorene)-alt-4,7-(2-(phenyl)benzo(d)(1,2,3) triazole)) bromide (HTMA-PFBT) (in green) and copoly-((9,9-bis(6′-*N,N,N*-trimethylammonium)hexyl)- 2,7-(fluorene)-alt-1,4-(naphtho(2,3c)-1,2,5-thiadiazole)) bromide (HTMA-PFNT) (in red).

Fluorene-based conjugated polyelectrolytes, being fluorescent, nontoxic and photostable, provide excellent thermal and chemical stability. Furthermore, fluorene-based CPEs have good synthetic accessibility at the C9 position of the fluorene ring. Polyfluorenes usually emit in the blue spectral region, but copolymerization with other aromatic units allows for shifting the emission spectrum to longer wavelengths [39,40,41]. On this matter, we have synthesized three cationic polyfluorenes which emit in the blue, green and red regions of the visible spectrum: copoly-((9,9-bis(6′-*N,N,N*-trimethylammonium)hexyl)-2,7-(fluorene)-alt-1,4-(phenylene)) bromide (HTMA-PFP), which incorporate a phenyl group on fluorene backbone, copoly-((9,9-bis(6′-*N,N,N*-trimethylammonium)hexyl)-2,7-(fluorene)-alt-4,7-(2-(phenyl) benzo(d) (1,2,3) triazole)) bromide (HTMA-PFBT), which incorporates the chromophore 2-phenylbenzotriazole on the backbone and copoly-((9,9-bis(6′-*N,N,N*-trimethylammonium)hexyl)-2,7-(fluorene)-alt-1,4-(naphtho(2,3c)-1,2,5-thiadiazole)) bromide (HTMA-PFNT), which incorporates a naphtha(2,3c) (1,2,5)thiadiazole group on fluorene backbone [42,43,44] (Scheme 1, upper part). The three CPEs have been extensively characterized, showing interesting properties as fluorescent membrane markers for bioimaging studies [42,43,44,45,46]. In addition, they have common absorption bands around 330–350 nm (Scheme 1, bottom part), therefore, they could be excited simultaneously upon UV excitation, allowing multicolor fluorescence imaging. Finally, because of their cationic charge, the synthesized CPEs have high affinity to anionic species, forming complexes with certain proteins and lipid vesicles [33,43].

In this work, we have taken advantage of the interesting properties of the fluorescent CPEs to develop a multifunctional nanoplatform able to integrate imaging and therapeutic functionalities in one entity. With this end, TSLs composed of the anionic lipid DPPG have been prepared and coated with HTMA-PFP, HTMA-PFBT and HTMA-PFNT, in order to obtain blue, green and red fluorescent drug carriers, respectively. The stability, size and morphology of the nanoparticles have been characterized, as well as their photophysical properties and thermotropic behavior. In addition, the suitability of the nanoparticles as carrier systems to release a drug in response to external mild hyperthermia has been explored using carboxyfluorescein (CF) as a model hydrophilic drug. Finally, preliminary experiments have been carried out to evaluate the capacity of the nanostructures to mark and visualize mammalian cells in different colors.

## 2. Materials and Methods

### 2.1. Materials

The synthetic phospholipid 1,2-dipalmitoyl-sn-glycero-3-phospho-rac-(1-glycerol) sodium salt (DPPG) was purchased from Sigma-Aldrich (St. Louis, MO, USA). The polyfluorenes HTMA-PFP (M_n_ (g·mol^−1^) = 4170; M_w_ (g·mol^−1^) = 8340), HTMA-PFBT (M_n_ (g·mol^−1^) = 4584; M_w_ (g·mol^−1^) = 8531) and HTMA-PFNT (M_n_ (g·mol^−1^) = 4507; M_w_ (g·mol^−1^) = 8990) were synthesized and subsequently characterized in our laboratory [43,44,47]. Stock solutions of the polyfluorenes were dissolved in dimethyl sulfoxide (DMSO) with a final concentration of 3.65 × 10^−4^ M for HTMA-PFP and HTMA-PFNT, and 6.24 × 10^−4^ M for HTMA-PFBT (in repeat units), and stored at −20 °C before use. The dye 5(6)-carboxyfluorescein (CF) was purchased from Sigma-Aldrich (St. Louis, MO, USA), as well as the fluorescent membrane probe 1,6-diphenyl-1,3,5-hexatriene (DPH), and the quencher 9,10-anthraquinone-2,6-disulfonic acid (AQS). Stock solutions of the three compounds were prepared in dimethyl sulfoxide (DMSO) (1.25 M), dimethylformamide (DFM) (1 mM) and water (5 mM), respectively. Phosphate buffer solution (50 mM, 0.1 M NaCl, pH 7.4) was prepared in Milli-Q water. The rest of the chemicals were of spectroscopic or analytical reagent grade.

### 2.2. Methods

#### 2.2.1. Preparation of Thermosensitive Liposomes (TSLs)

Lipids solutions of 2 mg of DPPG were left to dry at room temperature. Straight away, the dried phospholipid was resuspended in sodium phosphate buffer to a final concentration of 0.5 mM in order to obtain multilamellar vesicles (MLVs). MLVs were then heated above the phospholipid phase transition (~41 °C) and vortexed several times. TSLs were obtained from MLVs by pressure extrusion through 0.1 μm polycarbonate filters above T_m_ (~41 °C) in order to obtain homogenous large unilamellar vesicles (LUVs).

#### 2.2.2. Preparation of Multicolor Fluorescent Nanoparticles

**Scheme 2:**
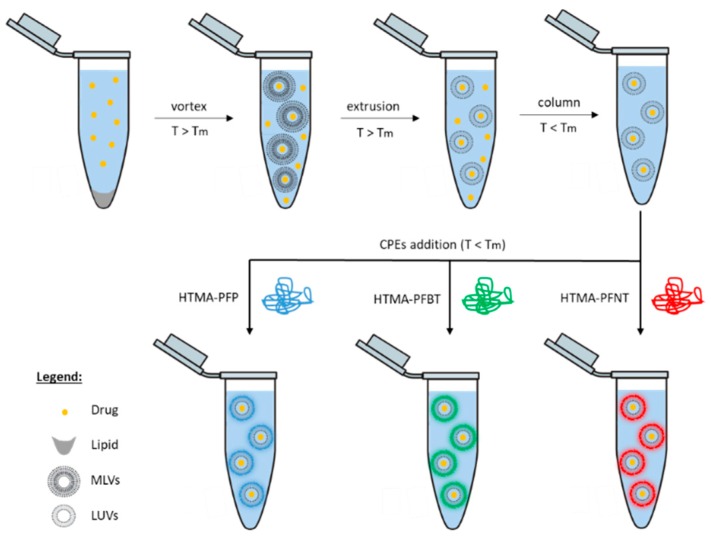
Preparation of multicolor fluorescent nanoparticles.

Aliquots of CPEs solubilized in DMSO (3.65 × 10^−4^ M for HTMA-PFP and HTMA-PFNT, and 6.24 × 10^−4^ M for HTMA-PFBT, in repeat units) were externally added to the TSLs suspension and incubated for at least 30 min at room temperature, as is depicted in Scheme 2. The proportion of DMSO in final samples was lower than 1% (*v*/*v*) in all the cases, and the final CPEs concentration was 3 μM in terms of repeat units.

#### 2.2.3. Drug Encapsulation and Release Assays

CF was used as a hydrophilic model drug to investigate the encapsulation and controlled release properties of the TSLs and multicolor fluorescent nanoparticles. Encapsulation of the compound was carried out as is depicted in Scheme 2. Briefly, TSLs composed of DPPG were prepared with CF encapsulated in the aqueous core at a concentration of 40 mM in sodium phosphate buffer. The non-encapsulated CF was taken off by using a gel filtration column loaded with Sephadex G-75 and eluted with sodium phosphate buffer. Aliquots of CPEs were externally added to the TSLs loaded with CF, as was previously described. Self-quenching is expected when the CF is entrapped inside the vesicles. The CF release was assayed by treating the CF-loaded vesicles with increasing temperatures up to 60 °C. Samples were excited at 492 nm in order to minimize the absorbance of CPEs, and the emission was collected between 500–550 nm. The amount of CF released during and after thermal treatment was determined by inducing the total breakdown of the vesicles with Triton X-100 at 10%.

#### 2.2.4. Particle Size and Zeta Potential

The size and Zeta Potential of the TSLs and multicolor fluorescent nanoparticles was explored by Dynamic Light Scattering (DLS) technique, with a Malvern Zetasizer Nano-ZS instrument (Worcestershire, UK) equipped with a monochromatic coherent 4 mW Helium Neon laser (λ = 633 nm) light source, where size measurements were performed at angles of 173°. Size was measured in disposable cuvettes, while Zeta Potential measurements were performed in specific Zeta Potential cells. All measurements were carried out in triplicate at room temperature.

#### 2.2.5. Fluorescence Experiments

Fluorescence measurements were carried out in a PTI-QuantaMaster Spectrofluorometer (Birmingham, AL, USA) equipped with a Peltier cell holder. 1 cm path length quartz cuvettes were used to place the samples, which were subsequently excited at 380 nm (HTMA-PFP), 510 nm (HTMA-PFNT), 425 nm (HTMA-PFBT) or 492 nm (CF). Background intensities were checked and removed from the samples when it was necessary.

#### 2.2.6. Anisotropy Experiments

Changes in the anisotropy as a function of temperature were used to explore the phase transition cooperativity and T_m_ of TSLs. Steady state anisotropy, <*r*>, is defined as:

(1) $$<r>=\frac{\left(I_{VV}-GI_{VH}\right)}{\left(I_{VV}+2GI_{VH}\right)}$$

where, *I_VV_* and *I_VH_* correspond to the fluorescence intensities collected with the excitation polarizer oriented in a vertical position, and the emission polarizer oriented in a vertical and horizontal position, respectively. These measurements were obtained using Glan–Thompson polarizers incorporated in the spectrofluorometer. The liposome samples in the presence of DPH were excited at 360 nm and the emission was collected at 430 nm. The G factor (*G* = $I_{HV}/I_{HH}$) corrects the transmissivity bias introduced by the equipment.

#### 2.2.7. Partition Coefficient Experiments

The partition coefficient, *Kp*, of the polyfluorenes between the gel-phase DPPG membranes of the TSLs and the aqueous medium was assayed by quantifying fluorescence intensity changes of CPEs in the presence of increasing TSLs concentrations. *K_p_* is defined as:

(2) $$K_{p}=\frac{n_{L}/V_{L}}{n_{w}/V_{w}}$$

where, *n_i_* and *V_i_* correspond with the moles and the volume of phase *i*, respectively. The phase *i* could be either lipidic (*i* = *L*) or aqueous (*i* = *W*). The determination of *K_p_* was carried out according to Reference [47]:

(3) $$\Delta I=\frac{\Delta I_{max}\left[L\right]}{1/\left(K_{p}\gamma \right)+\left[L\right]}$$

where, Δ*I* (Δ*I* = *I* − *I*_0_) corresponds with the difference between either fluorescence intensities or emission spectrum areas of the polyfluorenes measured in the presence (*I*) and absence (*I*_0_) of TSLs, Δ*I_max_* = *I*_∞_ − *I*_0_ represents the highest value of this difference once the lowest value is reached (*I*_∞_) upon increasing the TSLs concentration (*L*), and *γ* corresponds with the phospholipid molar volume (0.763 M^−1^) [48].

#### 2.2.8. Fluorescence Quenching Experiments

Fluorescence emissions of the CPEs in sodium phosphate buffer and integrated in TSLs were studied in the presence and absence of different AQS concentrations. This compound is an electron acceptor which works as a quencher of cationic CPEs, creating static quenching complexes through electrostatic interactions [49,50]. Stern–Volmer analysis was applied to the obtained fluorescence quenching values according to Equation (4):

(4) $$\frac{I_{0}}{I}=1+K_{SV}\left(Q\right)$$

where, *I* and *I*_0_ correspond with the steady-state fluorescence intensities in the presence and absence of AQS respectively, and (*Q*) represents the AQS concentration. The meaning of *K_SV_* relies on the nature of the quenching process: it could represent the rate of dynamic quenching or the association constant for complex formation (which is the case of AQS and CPEs) [51].

#### 2.2.9. Morphological Observation

The morphological observation of the TSLs and multicolor fluorescent nanoparticles was performed by using a transmission electron microscope (TEM) (JEM-1400 Plus, JEOL, Tokyo, Japan), working at 120 kV. A drop of the samples was placed on to 300-mesh copper grips coated with carbon. In order to visualize the vesicles, a drop of lead citrate was also added. Samples were left dry before being placed under the microscope. A Gatan ORIUS camera was employed to record the images.

#### 2.2.10. Cell Imaging Experiments

The human embryonic kidney cell line HEK293 was kindly donated by Dr. Alberto Falcó Gracia (Instituto de Investigación, Desarrollo e Innovación en Biotecnología Sanitaria de Elche, IDiBE, Elche, Spain). Cells were cultured in Dulbecco’s Modified Eagle Medium (DMEM) with 10% (*v*/*v*) fetal calfserum (FCS), 2 mM L-glutamine, 100 U/mL penicillin and 100 μg/mL streptomycin and maintained in an incubator with controlled humidity (5% CO_2_). 

For fluorescence microscopy experiments, 96-well plates were used. The final concentration was 10^5^ HEK293 cells per mL. Microscopy images were taken in the presence of multicolor fluorescent nanoparticles up to a final CPEs concentration of 0.365 µM, 0.73 µM and 0.18 µM of HTMA-PFP, HTMA-PFNT and HTMA-PFBT, respectively.

Fluorescence microscopy images were captured by using an inverted microscope (Leica DMI 3000B, Leica, Wetzlar, Germany) equipped with a compact light source (Leica EL6000) and a digital camera (Leica DFC3000G). The recording was carried out by using a 63× objective (0.7 magnification) and the filters: DAPI (Ex BP 350/50, Em BP 460/50), FITR (Ex BP 480/40, Em BP 527/30) or DsRed (Ex BP 555/25, Em BP 620/60). Data acquisition was performed manually with Leica Application Suite AF6000 Module Systems, and the image processing was carried out by using the software ImageJ.

## 3. Results

### 3.1. Characterization of Thermosensitive Liposomes (TSLs)

**Figure 1:**
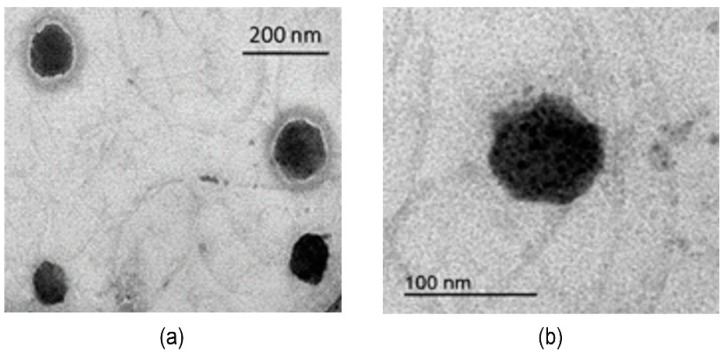
Transmission electron microscopy (TEM) images (**a**,**b**) of thermosensitive liposomes (TSLs) at different magnifications.

TSLs composed of DPPG were prepared as is described in the Methods Section (Section 2.2.1). Their size and stability were characterized by DLS and Zeta Potential, as shown in Table 1. The lipid was selected because its high ability to associate with cationic polielectrolytes as well as for its transition temperature in the mild hypertermia range. The experiments were done at 24 °C, where DPPG is in the gel phase. The vesicles exhibited hydrodynamic diameters around 130 nm and a high negative Zeta Potential, which should guarantee adequate suspension stability, since it was previously reported that Zeta Potential values higher than |20| mV are sufficient to prevent vesicle coalescence [52]. The polydispersity index value was 0.11, evidencing a narrow distribution of particle size. The morphology of the freshly prepared TSLs was observed under transmission electron microscopy. Results showed well-dispersed spherical-shaped vesicles, with a particle size compatible with the one estimated by DLS (Figure 1).

**Figure 2:**
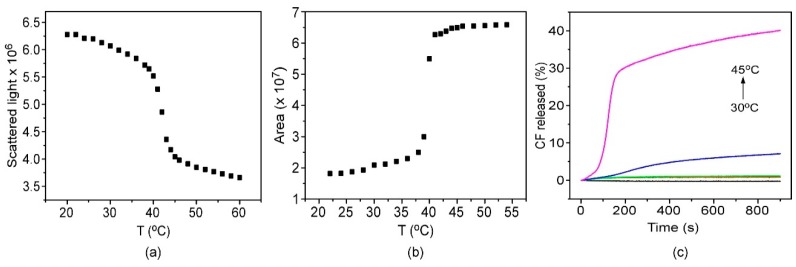
Effect of temperature on (**a**) the scattered light by TSLs and on (**b**) area of the emission spectrum of carboxyfluorescein (CF) encapsulated in TSLs in sodium phosphate buffer. (**c**) CF released in % as function of time (0–900 s) at different temperatures: 30 °C (black), 35 °C (red), 37 °C (green), 40 °C (blue) and 45 °C (magenta).3.2. Encapsulation and Release Assays.

The thermal behavior of the TSLs was explored using light scattering measurements. We analyzed the light scattered by the TSLs suspension as a function of temperature, taking into account that gel phase bilayers scatter more light than fluid membranes, a feature which has been attributed to the higher refractive index of gel membranes, as compared with fluid ones [53]. This experiment was directly made in the spectrofluorometer, by selecting the same wavelength for both excitation and emission monochromators with the smallest slit. The scattered light (430 nm) was collected at an angle of 90° of the incident light. As is shown in Figure 2a, a sharp drop of the scattered light was observed around 41–42 °C, which coincides with the transition temperature reported for DPPG. These results were confirmed by the fluorescence anisotropy measurements using the fluorescent probe DPH incorporated into the TSLs bilayer. This is a commonly used tool for thermotropic characterization of liposomes [54]. The plot of the steady-state fluorescence anisotropy, <*r*>, of DPH versus temperature is shown in the Supplementary Material (Figure S1), and was a perfect sinusoidal, displaying a sharp transition of anisotropy values around 42 °C. 

### 3.2. Encapsulation and Release Assays

The dye carboxyfluorescein (CF) was entrapped in the aqueous cavity of TSLs, as was described in the Methods Section (Section 2.2.3). This marker was chosen for the release assays because of its interesting photophysical properties. When CF is highly concentrated, most of its fluorescence is quenched due to dimerization to a nonfluorescent compound as well as the Förster Resonance Energy Transfer (FRET) process between monomers and dimers [55]. Therefore, it is possible to monitor its release from the TSLs measuring the increase in fluorescence as a function of temperature (Figure 2b) or time (Figure 2c). Figure 2b shows an abrupt increase in the fluorescence of CF above 40 °C, which evidences the release of the dye from the liposome close to the transition temperature. Samples were also exposed to temperatures ranging from 30 to 45 °C, and the time release profile was recorded at each temperature (Figure 2c). Every sample was preheated at 30 °C before being placed in the thermostatized fluorimeter holder. Percent release was calculated from the change in fluorescence intensity, as detailed in the Methods Section (Section 2.2.3), after addition of Triton X-100 10%. Results show that below 37 °C, the dye remains entrapped into the liposome, at least within the experimental time period. At 40 °C, it starts to be slowly removed from the TSLs, probably due to the coexistence of gel and fluid domains at temperatures just below T_m_, as has been reported in previous works for DPPG [56]. Finally, a rapid release of CF takes place at 45 °C, especially in the first three minutes, evidencing the ability of the DPPG-TSLs to carry and release hydrophilic compounds triggered by hyperthermia. The fact that only a 40% of CF is released at this temperature after 15 min of incubation could be attributed to dye interaction with the lipid membrane [56].

### 3.3. Preparation and Characterization of Fluorescent Nanoparticles

Once the physical properties and thermal behavior of the TSLs were characterized, the next step was to obtain the fluorescent nanoparticles. They were prepared by incorporation of the three polyelectrolytes: blue (HTMA-PFP), green (HTMA-PFBT) and red (HTMA-PFNT) in the bilayer of TSLs, at 25 °C (gel phase), as is shown in Scheme 2. This temperature was selected to prevent the release of the encapsulated drug from the TSLs before heating. In previous works, we demonstrated the ability of these CPEs to interact and insert into fluid phase lipid bilayers, by determining their affinity and membrane location [42,43,44]. But, taking into account the higher lipid packing of the gel phase, we cannot assume that the amount of polyelectrolyte bound to the fluid-phase membrane was the same as that bound to the gel-phase membrane. Therefore, the first experiments were focused to estimate the affinity of the CPEs for the TSLs, by determining their partition coefficient, K_p_, defined in Equation (2). With this end, three series of samples containing increasing concentrations of DPPG-TSLs with final lipid concentrations ranging from 0 to 1 mM were prepared in buffer, and a constant concentration (3 µM) of HTMA-PFP, HTMA-PFBT and HTMA-PFNT was added to each series of samples respectively, which were incubated for 30 min at room temperature. 

**Figure 3:**
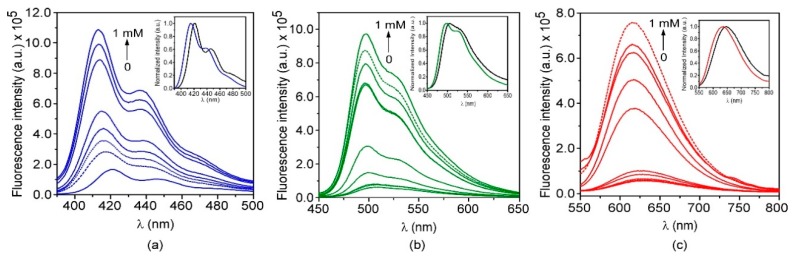
Fluorescence emission spectra of (**a**) HTMA-PFP (3 µM), (**b**) HTMA-PFBT (3 µM) and (**c**) HTMA-PFNT (3 µM) in buffer with increasing concentrations of TSLs. Insets: Normalized fluorescence emission spectra of CPEs in sodium phosphate buffer (black) and incorporated in TSLs (color).

The emission spectra for the three polyelectrolytes, recorded at the different lipid concentrations, are shown in Figure 3a–c. A low fluorescence emission signal was detected for the CPEs in buffer, as a consequence of the formation of metastable aggregates [42,43,44]. The increase of the lipid concentration induced an enhancement in fluorescence intensity and a blue-shift of the emission spectra (insets in Figure 3), which suggests the breaking of aggregates as a consequence of the interaction of the three polyelectrolytes with TSLs-bilayer. Plotting the area of each spectrum versus lipid concentration (Figure S2) and using Equation (3), it was possible to determine the K_p_ values for the three CPEs. These values are summarized in Table 2 and indicate that, although the bilayer is in the gel-phase, the three polymers have high affinity for the TSLs. However, the K_p_ values are one order of magnitude lower than those obtained for anionic membranes in the fluid-phase [42,43,44]. This result suggests that the mode and nature of the interaction between CPEs and lipid membranes is different in the two phases. Probably, in the gel-phase, the interaction is mainly electrostatic between the quaternary amine groups of CPEs and the negative charge of the lipid head groups. In contrast, in the fluid-phase, the decrease of the lipid packing allows the insertion of the polymer chains and the hydrophobic forces contribute to a better solubilization. The fact that the fluorescence intensity of the CPEs incorporated in the gel-phase was lower than that registered in the fluid-phase at the same conditions (data not shown), supports this hypothesis. 

The K_p_ values were used to optimize the concentration of each component for the fabrication of the fluorescent nanoparticles. The lipid concentration was fixed to 0.5 mM to limit the turbidity of the samples, which becomes an obstacle for fluorescence measurements. The concentration of the CPEs was 3 µM in order to obtain a good fluorescent signal and to ensure that more than 90% of the polyelectrolyte was bound to the TSLs. The stability of the nanoparticles was assessed by monitoring the fluorescence intensity of the sample in the maximum of the emission spectrum as a function of time, after addition of the CPEs. The signal stabilized in the first seconds for HTMA-PFP and HTMA-PFNT and in ~5 min for HTMA-PFBT and remained stable during the experimental time (Figure S3). In addition, the possibility of simultaneously exciting the fluorescence emission of blue, green and red nanoparticles suspended in the same sample was also studied. The mixture was excited at 335 nm, where the three polyelectrolytes absorb (see Scheme 1). The recorded spectrum, shown in Figure S4, clearly displays the three bands corresponding to the characteristic spectra of HTMA-PFP, HTMA-PFBT and HTMA-PFNT.

**Figure 4:**
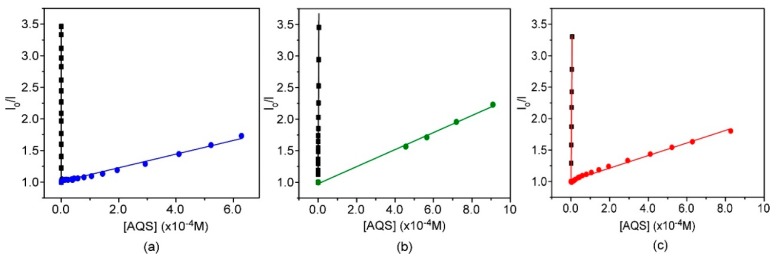
Stern–Volmer plots for quenching of (**a**) HTMA-PFP (3 µM), (**b**) HTMA-PFBT (3 µM) and (**c**) HTMA-PFNT (3 µM) by AQS in sodium phosphate buffer (squares) and in TSLs (circles).

The localization of the CPEs in the lipid bilayer of TSLs was investigated by quenching experiments using the anionic acceptor AQS as a fluorescence quencher. This molecule has been observed to be an excellent quencher for cationic conjugated polyelectrolytes, and it is soluble in water but not in lipid bilayer, so the fluorescence only will be deactivated if the CPEs are in the buffer or in the membrane surface [43,46]. When increasing concentrations of AQS were added to three different samples, containing the three polyelectrolytes in buffer, a strong decrease in their fluorescence signal was observed. In contrast, when the same experiment was performed in samples containing the fluorescent nanoparticles, the quenching effect was much less efficient (Figure 4). The Stern–Volmer plots (Equation (4)) were linear in all the studied ranges, with K_sv_ values similar in the three multicolor fluorescent nanoparticles, ranging from 1.00 × 10^3^ M^−1^ for red fluorescent nanoparticles to 1.32 × 10^3^ M^−1^ for green fluorescent nanoparticles. These quenching values are lower than in buffer (Table 2), confirming that the polyelectrolytes are bound to the lipid bilayer but close to the surface, because they are relatively accessible to the quencher. Finally, we compared the K_sv_ values obtained in the fluorescent nanoparticles with those achieved in anionic (PG) lipid vesicles in fluid-phase, which were ~0, especially for HTMA-PFP and HTMA-PFNT [46]. These differences in K_sv_ support the hypothesis previously proposed that the lipid packing affects the mode of interaction between the polyelectrolytes and the lipid membrane, as well as their final location in the bilayer. 

**Figure 5:**
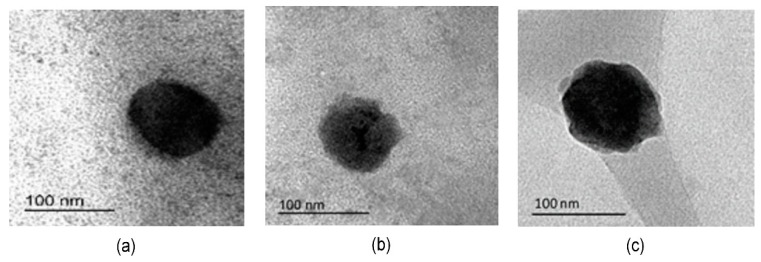
Transmission electron microscopy (TEM) images of blue (**a**), green (**b**) and red (**c**) fluorescent nanoparticles.

Once optimized and analyzed, the distribution of components of the fluorescent nanoparticles we characterized by their size and colloidal stability, as well as their morphology (Table 1 and Figure 5). DLS results show that the incorporation of the polyelectrolytes slightly increases the size of the TSLs, which is compatible with their location close to the membrane surface. The decrease in the Zeta Potential was minimum and the nanoparticles exhibited good colloidal stability, preserving their spherical shape. 

**Figure 6:**
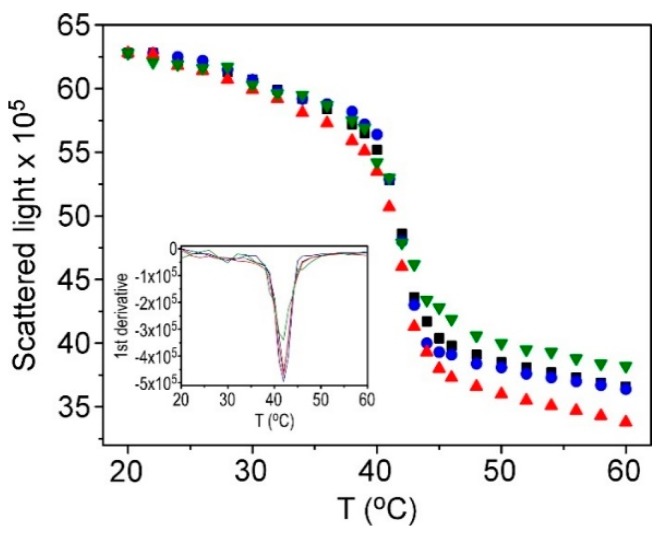
Effect of temperature on the light scattered by TSLs (squares) and by blue (circles), green (down-triangles) and red (triangles) fluorescent nanoparticles. Inset: First derivative of the thermograms.

The evaluation of the thermosensitive properties of the fluorescent nanoparticles were carried out through two kinds of studies. First, we explored if the incorporation of CPEs modified the thermotropic behavior of the TSLs, previously characterized in Figure 2, and second, we analyzed if the fluorescence of the polyelectrolytes was sensitive to the structural modifications which take place in the vesicle bilayers at the lipid phase transition. For the first purpose, light scattering measurements were performed on the nanoparticles’ suspension, as a function of temperature. Figure 6 shows the thermograms recorded for the fluorescent nanoparticles, blue, green and red, as well as for the TSLs in the absence of CPEs. The shape of the obtained curves is similar in the four samples: the light scattered by the nanoparticles slightly decreases as temperature rises, with a sharp drop occurring at T_m_, whose value can be obtained from the first derivative plot and is coincident with that obtained in the absence of CPEs (inset in Figure 6). These results indicate that the integration of the polyelectrolytes does not affect the T_m_ and cooperativity of the lipid transition and thus, the fluorescent nanoparticles display the same thermosensitive properties as the DPPG-TSLs. 

**Figure 7:**
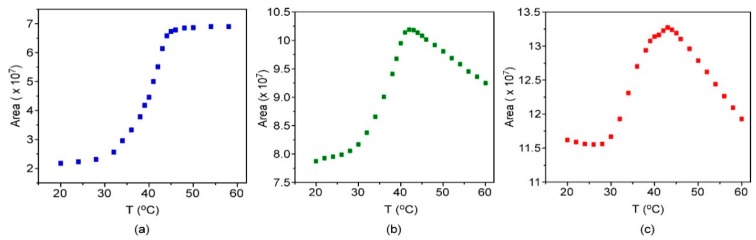
Effect of temperature on the emission spectrum area of (**a**) blue, (**b**) green and (**c**) red fluorescent nanoparticles.

As for the second purpose, the emission spectra of the three fluorescent nanoparticles were recorded as a function of temperature and the area under each spectrum was plotted between 20 and 60 °C (Figure 7a–c). In these plots, it is possible to distinguish three regions with a different behavior. Between 20 and 32 °C, the changes in fluorescence intensity are not very noticeable. However, above 30 °C, a rise in the fluorescence intensity is observed, reaching a maximum value around 42–45 °C. One possible explanation for this behavior could be that the CPEs are sensitive to the lipid pretransition, which occurs in DPPG at temperatures above 30 °C. The lipid pretransition is a transition of low enthalpy occurring below T_m_, in which a flat bilayer in gel-phase becomes a periodically undulated membrane, called the ripple-phase [56]. In this ripple-phase, the hydrocarbon chains remain mainly in their rigid, extended, all-trans conformation, like in the gel-phase. However, some works have proposed the possible existence of fluid regions coupled with the geometry of the ripples [56]. 

Probably, when the CPEs are added to the DPPG-TSLs at 24 °C, they are mainly adsorbed to the surface instead of being incorporated in the lipid bilayer, as is demonstrated from the quenching experiments. The beginning of pretransition, above 30 °C, allows the polyelectrolytes to go into the lipid membrane, which leads to an increase of fluorescence emission intensity that reaches its plateau close to T_m_. Once the fluid phase is reached, the polyelectrolye remains embedded in the bilayer and the fluorescence signal stabilizes or tends to decrease because the probability of nonradiative transitions increases with increasing temperature.

### 3.4. Nanoparticles as Drug Carriers and Bioimaging Probes

**Figure 8:**
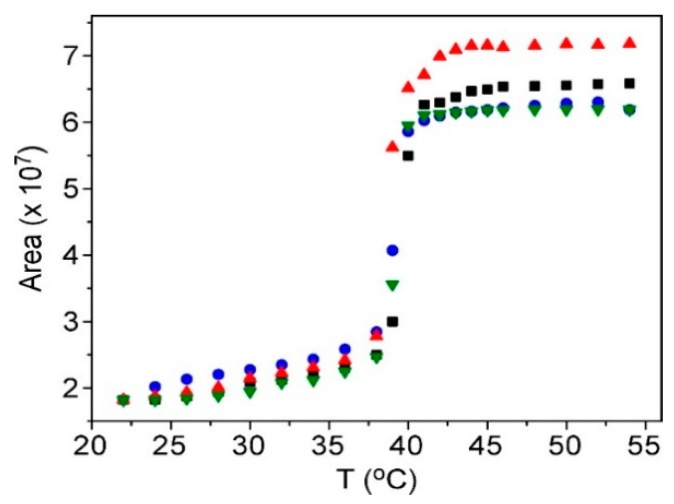
Area of the emission spectrum of CF encapsulated in TSLs (squares) and blue (circles), green (down-triangles) and red (triangles) fluorescent nanoparticles as function of temperature (20–55 °C).

**Figure 9:**
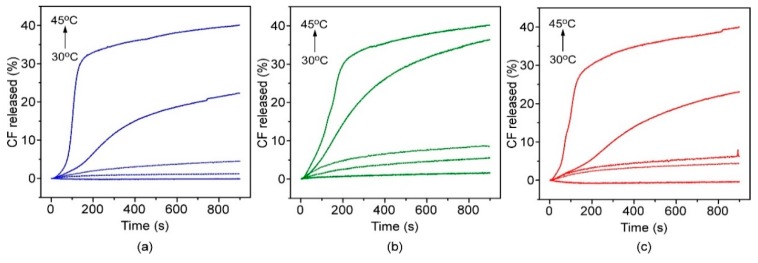
CF released in % as function of time (0–900 s) at different temperatures (30, 35, 37, 40 and 45 °C) in blue (**a**), green (**b**) and red (**c**) fluorescent nanoparticles multicolor fluorescent nanoparticles.

The above experiments indicate that the fluorescent nanoparticles preserve the thermosensitive properties of the TSLs, but they do not inform if the ability to encapsulate and release hydrophilic compounds triggered by hyperthermia is maintained. In fact, the possible internalization of the CPEs in the lipid membrane above 32 °C (as previously suggested), could cause a disruption of the lipid packaging, causing the compounds to be released from the nanoparticle before reaching T_m_. To check this possibility, CF was firstly entrapped in the aqueous cavity of TSLs and the CPEs were then added to the suspension, as is described in Scheme 2. To monitor the release of CF from the nanoparticles, we recorded the fluorescence intensity of the dye as a function of temperature (Figure 8). The profile of the curves in Figure 8 was very similar for blue, green and red nanoparticles as well as for TSLs in the absence of CPEs. Above 40 °C, an abrupt increase in the fluorescence intensity of CF was detected, which suggests that most of the dye is released from the nanoparticles when the phase transition is reached. However, to confirm this conclusion, it is necessary to perform release kinetics of the dye at different temperatures. Nanoparticles were then exposed to temperatures ranging from 30 to 45 °C and the time release profile was recorded at each temperature (Figure 9), as was previously performed for the TSLs suspension. Results indicate that the release kinetics from the nanoparticles presents some differences with respect to those obtained in the absence of CPEs (Figure 2c). The most significant difference is that in the case of nanoparticles, an important fraction of CF is released at 40 °C, especially from the green nanoparticles. In addition, ~5% of the dye is released at 37 °C, after different incubation periods, depending on the fluorescent nanoparticles. For the blue one, it was necessary to have more than 900 s of incubation, while for the green and red nanoparticles, the release percentage of 5% was reached at 200 and 400 s, respectively. Therefore, although Figure 8 suggests that the presence of CPEs is not affecting the release properties of the TSLs, the internalization of the polyelectrolytes, which takes place above the pretransition temperature, seems to slightly affect the permeability of the membrane, allowing a small fraction of the dye to be slowly released at temperatures below T_m_.

**Figure 10:**
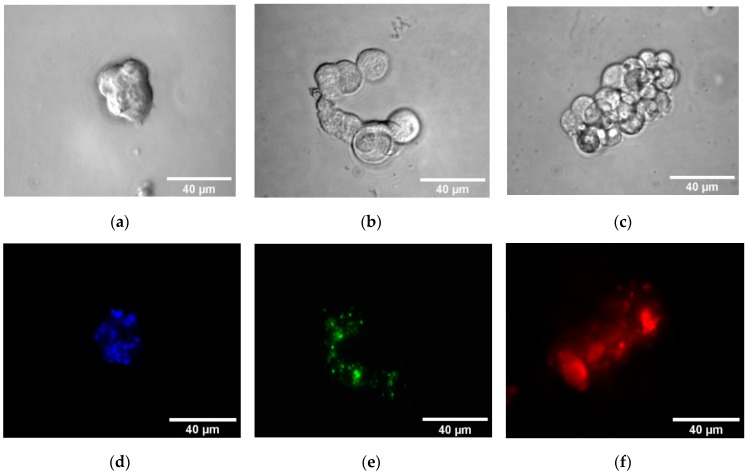
Microscopy images of HEK293 cells in the presence of (**a**, **d**) blue, (**b**, **e**) green and (**c**, **f**) red fluorescent nanoparticles, observed under (**a**–**c**) phase contrast and (**d**–**f**) visible-light using the Leica DAPI filter (Ex BP 350/50, Em BP 460/50), DsRed filter (Ex BP 555/25, Em BP 620/60) and FITR filter (Ex BP 480/40, Em BP 527/30).

Finally, we have performed preliminary experiments to test the capability of the fluorescent nanoparticles to be employed as bioimaging probes. With this end, phase contrast and fluorescence microscopy images of HEK293 cells were taken before and 30 min after the addition of blue, green and red nanoparticles. Figure 10 shows the HEK293 cells in the presence of the polyfluorene-based fluorescent nanoparticles, observed by phase contrast and fluorescent microscopy. Phase contrast and fluorescence images correspond to the same field for each type of fluorescent nanoparticle. These images clearly show that nanoparticles are able to interact with cells, allowing for their visualization in three different colors under fluorescence microscopy. This result extends the applications of these new fluorescent nanoparticles, which could be used as probes for bioimaging while transporting and monitoring the pathway of a drug, controlling its release.

## 4. Conclusions

Blue, green and red fluorescent nanoparticles composed of thermosensitive liposomes (TSLs) of DPPG and the conjugated polyelectrolytes HTMA-PFP, HTMA-PFBT and HTMA-PFNT respectively, have been prepared and characterized in order to obtain fluorescent drug carriers. In addition, the ability of the nanoparticles for bioimaging applications, transport and control drug delivery has been explored, evidencing their potential use as multifunctional nanoplatforms with imaging and therapeutic functionalities.

The nanoparticles exhibited stable fluorescence signals, good colloidal stability, spherical morphology and hydrodynamic diameters slightly higher to those of DPPG-TSLs, suggesting a membrane surface location of polyelectrolytes, which was confirmed by quenching experiments. In addition, their thermosensitive properties (cooperativity and transition temperature close to 42 °C) were similar to those of the DPPG-TSLs, supporting the potential use of these nanoparticles to carry and release drugs triggered by mild hyperthermia. The use of the dye carboxyfluorescein (CF) as a model hydrophilic drug allowed confirmation of this assumption. The dye was entrapped in the aqueous cavity of the fluorescent nanoparticles and was mostly released when nanoparticles were incubated above 40 °C. However, a small fraction of dye was slowly released at temperatures near 37 °C. This behavior has been attributed to a deeper penetration of the polyelectrolytes in the lipid bilayer, occurring above the pre-transition temperature, which could slightly modify the membrane permeability, allowing the slow release of the dye.

Finally, preliminary experiments with mammalian cells showed the capability of the nanoparticles to mark and visualize cells in blue, green and red colors, extending their applications as bioimaging probes. In this respect, it would be possible to encapsulate a different hydrophilic drug in each type of nanoparticle. This result could be of great interest in two-photon excitation microscopy and for dynamic imaging of living cells, due to the possibility of simultaneously exciting the fluorescence emission of the three nanoparticles at a single wavelength. We plan to further expand the multifunctionality of the nanoparticles in the future by linking different tumor-targeting molecules, such as peptides, folic acid, antibodies or other small molecules to the fluorescent lipid vesicles. By this procedure, the nanoparticles can be more effectively targeted in order to perform differential cell marking and active delivery to different tumor sites, which could be visualized in real time.

## Figures and Tables

**Table 1:** Hydrodynamic diameter (d) and Zeta Potential (ZP) of TLSs and blue, green and red fluorescent nanoparticles.

	TSLs	TSLs + HTMA-PFP	TSLs + HTMA-PFBT	TSLs + HTMA-PFNT
d (nm)	127.3 ± 1.3	133.4 ± 0.9	143.5 ± 0.5	143.1 ± 0.1
ZP (mV)	−35.2 ± 1.7	−32.0 ± 0.7	−28.3 ± 0.7	−30.9 ± 0.9

**Table 2:** Partition coefficient, K_p_, values, and Stern–Volmer constants, K_SV_, for HTMA-PFP, HTMA-PFBT and HTMA-PFNT in TSLs by using 9,10-anthraquinone-2,6-disulfonic acid (AQS) as a quencher.

	K_p_	K_SV_ (M^−1^)
HTMA-PFP + TSLs	3.04 ± 0.66 × 10^4^	1.11 ± 0.02 × 10^3^
HTMA-PFP + Buffer		1.60 + 0.01 × 10^7^
HTMA-PFBT + TSLs	5.03 ± 1.16 × 10^4^	1.32 ± 0.02 × 10^3^
HTMA-PFBT + Buffer		6.08 ± 0.34 × 10^5^
HTMA-PFNT + TSLs	2.59 ± 0.41 × 10^4^	1.00 ± 0.02 × 10^3^
HTMA-PFNT + Buffer		4.19 ± 0.04 × 10^5^

## Supplementary Materials

The following are available online at https://www.mdpi.com/2079-4991/9/10/1485/s1, Figure S1. Anisotropy values, <*r*>, of DPH in DPPG-TSLs as function of temperature (20–70 °C) in sodium phosphate buffer, Figure S2. Changes in fluorescence intensity (Δ*I*) of (a) HTMA-PFP (3 µM), (b) HTMA-PFBT (3 µM) and (c) HTMA-PFNT (3 µM) at increasing concentrations of DPPG, Figure S3. Stability kinetics of (a) blue, (b) green and (c) red fluorescent nanoparticles (squares) compared with the stability of the corresponding polyelectrolytes in sodium phosphate buffer (circles), measured at 25 °C by monitoring their fluorescence intensity (blue: λexc = 380 nm, λem = 412 nm; green: λexc = 425 nm, λem = 500 nm; red: λexc = 510 nm, λem = 622 nm), Figure S4. Fluorescence emission spectrum of a sample containing simultaneously blue, green and red nanoparticles, upon excitation at 335 nm.
