# Supplementary material for: Polyfluorene-Based Multicolor Fluorescent Nanoparticles Activated by Temperature for Bioimaging and Drug Delivery
Source: Nanomaterials (Basel). 2019 Oct 18;9(10):1485. doi: 10.3390/nano9101485 (PMC6835524; doi:10.3390/nano9101485)
Supplement: Supplementary file 1 [file nanomaterials-09-01485-s001.pdf]

Supplementary Material

Polyfluorene-Based Multicolor Fluorescent Nanoparticles Activated by Temperature for Bioimaging and Drug Delivery

Marta Rubio-Camacho, Yolanda Alacid, Ricardo Mallavia, María José Martínez-Tomé *, and C. Reyes Mateo *

Instituto de Investigación Desarrollo e Innovación en Biotecnología Sanitaria de Elche (IDiBE), Universidad Miguel Hernández de Elche (UMH), 03202 Elche, Alicante, Spain; marta.rubioc@umh.es (M.R.-C.); yoli2395@gmail.com (Y.A.); r.mallavia@umh.es (R.M.)

* Correspondence: mj.martinez@umh.es (M.J.M.-T.); rmateo@umh.es (C.R.M.); Tel.: +34-966-652-475 (M.J.M.-T.); +34-966-658-469 (C.R.M.)

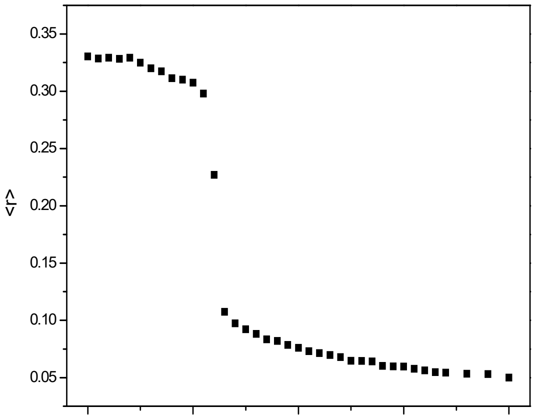

Figure S1. Anisotropy values, <r>, of DPH in DPPG-TSLs as function of temperature (20–70ºC) in sodium phosphate buffer.

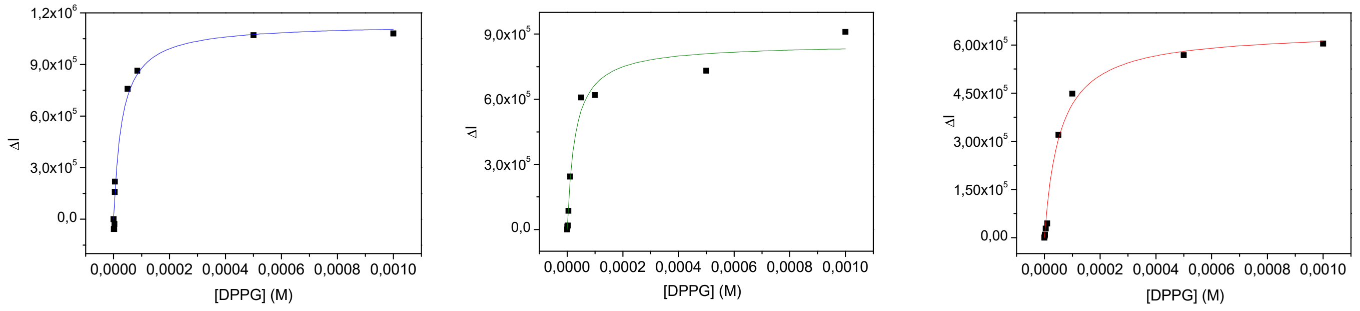

(a) **(b)** **(c)**

Figure S2. Changes in fluorescence intensity (ΔI) of (**a**) HTMA-PFP (3 μM), (**b**) HTMA-PFBT (3 μM) and (**c**) HTMA-PFNT (3 μM) at increasing concentrations of DPPG.

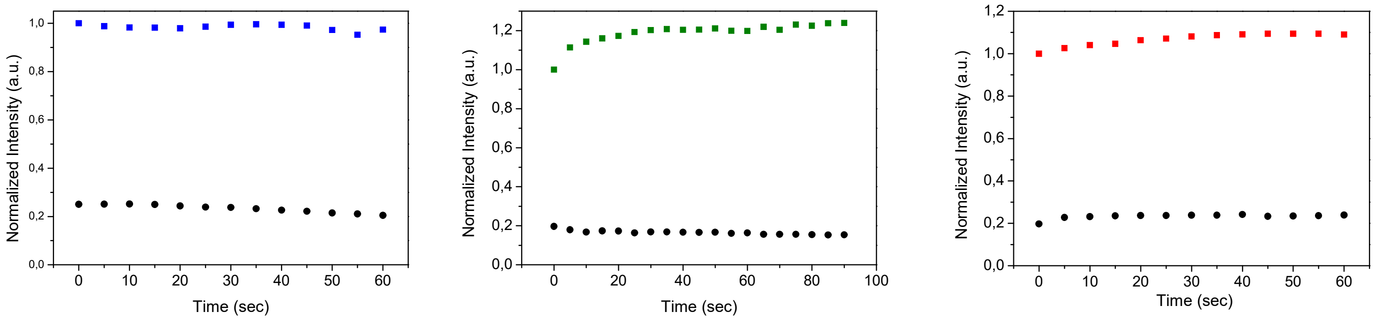

(a) **(b)** **(c)**

Figure S3. Stability kinetics of (**a**) blue, (**b**) green and (**c**) red fluorescent nanoparticles (squares) compared with the stability of the corresponding polyelectrolytes in sodium phosphate buffer (circles), measured at 25 ºC by monitoring their fluorescence intensity (blue: λexc = 380nm, λem = 412nm; green: λexc = 425nm. λem = 500 nm; red: λexc = 510 nm, λem = 622nm).

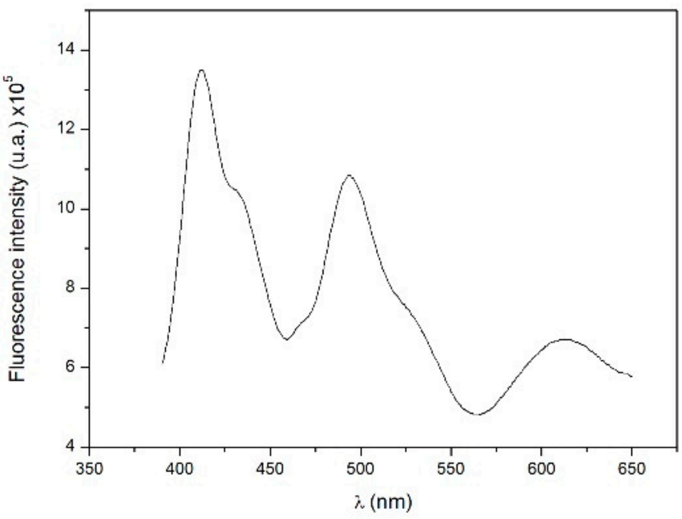

Figure S4. Fluorescence emission spectrum of a sample containing simultaneously blue, green and red nanoparticles, upon excitation at 335 nm.
